# Editorial: Brain cancer pathogenesis and data integration

**DOI:** 10.3389/fgene.2023.1298285

**Published:** 2023-10-13

**Authors:** Andrea Comba, Xinzhong Li, Barbara Breznik

**Affiliations:** ^1^ Division of Neuropathology, Department of Pathology, Heersink School of Medicine and the O'Neal Comprehensive Cancer Center, University of Alabama at Birmingham, Birmingham, AL, United States; ^2^ School of Health and Life Sciences, Teesside University, Middlesbrough, United Kingdom; ^3^ Department of Genetic Toxicology and Cancer Biology, National Institute of Biology, Ljubljana, Slovenia

**Keywords:** brain tumor, data integration, therapeutic resistance, biomarkers, liquid biopsies

Brain tumors are one of the most aggressive malignancies in humans. They can be classified as primary tumors, which arise in the brain, or secondary tumors, which arise elsewhere in the body and initially metastaze the brain. The morbidity and mortality of brain tumors is one of the highest among cancers (Siegel et al., 2023). Of particular concern is that mortality and incidence of brain tumors are increasing, especially in the population under 44 years of age. Brain tumor mortality in this population is 13.4%. For example, primary brain tumors are the most common cancer in children and the leading cause of death in pediatric cancer patients (Gould, 2018). Considering the low survival rate of adult and pediatric brain tumor patients and the detrimental impact on patient quality of life, economic costs, and mortality rates, there is an urgent need to develop more effective therapeutic approaches. Despite major research efforts, there are currently no effective treatment modalities or prevention strategies that would significantly improve the quality of life and disease outcome of brain tumor patients.

This Research Topic is a collection of original research and review articles that provide a deeper understanding of brain tumor progression and its heterogeneity in order to develop new patient-oriented treatment strategies. In addition, new methods such as database bioinformatics and liquid biopsy analyzes are used and discussed to complement traditional methods in the search for better treatment.

Epigenetic modifications of DNA and RNA expression are crucial for brain tumor development and their clinical classification (Capper et al., 2018). However, there are still some gaps in our knowledge how and which epigenetic modifications are involved in brain tumor treatment resistance and how we can use them for clinical decision making. In their original research study, Zhang et al. identified prognosis-related m6A regulatory genes that could be responsible for poor prognosis and serve as reliable biomarkers for glioma patient survival. Expression of these genes was also associated with genomic alterations. The m6A regulatory genes may serve as both reliable biomarkers and potential targets to improve glioma patient survival.

Notch signaling is involved in multiple cellular processes and glioma progression (Dell’Albani et al., 2014), but the application of Notch receptors in clinical practice in primary glioblastoma, the most aggressive primary brain tumor in adults, remains to be elucidated. In the study by Zheng et al., Notch3 was identified as an independent prognostic factor for primary glioblastoma in cohorts from publicly available databases and was closely related to immune cell infiltration and tumor cell proliferation. This study serves as the basis for further studies to clarify the precise role of Notch3 in glioblastoma progression and to analyze Notch3 for clinical benefit to patients.

The study by Jiang et al. identified a signaling molecule with GTPase activity from the Rho family CDC42 (Melendez et al., 2011) as a promising immunological target in glioma. Analysis of publicly available databases including TCGA, CGGA, TIMEER, GTEx and CPTAC revealed a strong association of CDC42 with poor glioma prognosis and a correlation with inflammatory responses in gliomas associated with immunosuppression in tumors. This suggests that CDC42 may be a target for immunotherapy of gliomas as well as other tumors.

New technologies are constantly being developed for more efficient and less invasive diagnosis and monitoring of response to treatment of brain tumors. Brain biopsies remain the gold standard for the diagnosis of pediatric brain tumors, but it is a high-risk procedure, especially for children. Monitoring liquid biopsies, such as those from CSF or blood, may provide a less invasive and more sensitive approach to diagnosing and monitoring response to treatment (Cantor et al., 2022). The review article by Tripathy et al. summarizes recent advances in the diagnosis and response to treatment of children with primary brain tumors using the latest technologies for liquid biopsy analysis. The authors provide an overview of biofluid collection, extraction, detection, and diagnosis to improve treatment outcomes. With the increased sensitivity of sequencing and other technologies to detect biomarkers in blood samples, the focus is shifting from CSF to serum testing in patients. In this direction, further research is needed to characterize and standardize biomarker detection in liquid biopsies, which may represent a shift in clinical decision making in pediatric brain tumors.

Taken together, the Research Topic *Brain Cancer Pathogenesis and Data integration* provides several new insights and biomarkers for brain cancer pathogenesis, as well as novel methodologies that have the potential to improve clinical decision making and treatment of brain cancer patients in the future.
